# Systemic oxidative stress is associated with lower aerobic capacity and impaired skeletal muscle energy metabolism in heart failure patients

**DOI:** 10.1038/s41598-021-81736-0

**Published:** 2021-01-26

**Authors:** Takashi Yokota, Shintaro Kinugawa, Kagami Hirabayashi, Mayumi Yamato, Shingo Takada, Tadashi Suga, Ippei Nakano, Arata Fukushima, Shouji Matsushima, Koichi Okita, Hiroyuki Tsutsui

**Affiliations:** 1 Department of Cardiovascular Medicine, Faculty of Medicine and Graduate School of Medicine, Hokkaido University, Sapporo, Japan; 2 Innovation Center for Medical Redox Navigation, Kyusyu University, Fukuoka, Japan; 3 Department of Cardiovascular Medicine, Faculty of Medicine and Graduate School of Medicine, Kyusyu University, Fukuoka, Japan; 4 Graduate School of Program in Lifelong Learning Studies, Hokusho University, Ebetsu, Japan; 5 Clinical Research and Medical Innovation Center, Hokkaido University Hospital, Kita-14, Nishi-5, Kita-Ku, Sapporo, 060-8648 Japan

**Keywords:** Cardiology, Diseases, Health care, Medical research

## Abstract

Oxidative stress plays a role in the progression of chronic heart failure (CHF). We investigated whether systemic oxidative stress is linked to exercise intolerance and skeletal muscle abnormalities in patients with CHF. We recruited 30 males: 17 CHF patients, 13 healthy controls. All participants underwent blood testing, cardiopulmonary exercise testing, and magnetic resonance spectroscopy (MRS). The serum thiobarbituric acid reactive substances (TBARS; lipid peroxides) were significantly higher (5.1 ± 1.1 vs. 3.4 ± 0.7 μmol/L, *p* < 0.01) and the serum activities of superoxide dismutase (SOD), an antioxidant, were significantly lower (9.2 ± 7.1 vs. 29.4 ± 9.7 units/L, *p* < 0.01) in the CHF cohort versus the controls. The oxygen uptake (VO_2_) at both peak exercise and anaerobic threshold was significantly depressed in the CHF patients; the parameters of aerobic capacity were inversely correlated with serum TBARS and positively correlated with serum SOD activity. The phosphocreatine loss during plantar-flexion exercise and intramyocellular lipid content in the participants' leg muscle measured by ^31^phosphorus- and ^1^proton-MRS, respectively, were significantly elevated in the CHF patients, indicating abnormal intramuscular energy metabolism. Notably, the skeletal muscle abnormalities were related to the enhanced systemic oxidative stress. Our analyses revealed that systemic oxidative stress is related to lowered whole-body aerobic capacity and skeletal muscle dysfunction in CHF patients.

## Introduction

Exercise intolerance is a cardinal symptom of chronic heart failure (CHF), leading to increased morbidity and mortality as well as a poor quality of life^1^. Reduced whole-body aerobic capacity in particular, characterized by a lower peak oxygen uptake (peak VO_2_) and lower VO_2_ at anaerobic threshold (AT VO_2_), is known as an independent predictor of all-cause mortality in CHF patients^2,3^. Among the various contributors to exercise tolerance, skeletal muscle function is a major determinant of the whole-body aerobic capacity as the skeletal muscles are the largest energy production sites during exercise. We and others have shown that abnormal skeletal muscle energy metabolism is responsible for the exercise intolerance in patients with CHF^4–6^.

Oxidative stress is generally caused by an imbalance between the production of reactive oxygen species (ROS) and the antioxidant defense capacity. Systemic oxidative stress has been shown to have prognostic value and is associated with heart failure (HF)-related symptoms evaluated by the New York Heart Association (NYHA) functional class in CHF patients^7–9^. However, the NYHA functional class is a subjective estimation of a patient's functional ability, and it is thus necessary to determine the roles and effects of systemic oxidative stress with a more objective estimation of functional capacity.

We conducted the present study to determine whether systemic oxidative stress evaluated by serum lipid peroxidation products and the antioxidant defense capacity is associated with lower whole-body aerobic capacity and with impaired skeletal muscle energy metabolism in patients with CHF.

## Results

### Patient characteristics

The baseline characteristics of the CHF and control groups are summarized in Table 1. There were no significant differences in age or any anthropometric parameters between the CHF and control groups. Of the 17 patients with CHF, 13 patients had dilated cardiomyopathy and four patients had other heart diseases including valvular heart disease and hypertensive heart disease. The rate of NYHA functional classes I, II, and III in the CHF group were 24%, 41%, and 35%, respectively. The fasting blood glucose, plasma insulin, homeostasis assessment model of insulin resistance (HOMA-IR), glycated hemoglobin (HbA1c), low-density lipoprotein (LDL)-cholesterol, triglycerides, and free fatty acids were similar between the CHF and control groups. The high-density lipoprotein (HDL)-cholesterol level was significantly lower in the CHF group compared to the control group. The peak VO_2_ and the AT VO_2_ were significantly decreased in the CHF group compared to the control group, indicating lower whole-body aerobic capacity in the CHF patients.

**Table 1:** Characteristics of the control subjects and CHF patients.

	Control (n = 13)	CHF (n = 17)	*p* value
Age (years)	48 ± 8	51 ± 12	0.35
Body weight (kg)	68.3 ± 9.4	64.1 ± 7.3	0.18
BMI (kg/m^2^)	23.4 ± 2.6	22.9 ± 2.8	0.68
Waist circumference (cm)	82 ± 7	85 ± 8	0.25
**NYHA functional class**			
I	–	4 (24)	n.a
II	–	7 (41)	n.a
III	–	6 (35)	n.a
**Blood biochemistry**			
Fasting blood glucose (mmol/L)	5.1 ± 0.5	5.4 ± 0.7	0.19
Insulin (pmol/L)	30.5 ± 12.6	35.8 ± 14.0	0.32
HOMA-IR	1.0 ± 0.4	1.2 ± 0.6	0.22
HbA1c (%)	5.1 ± 0.3	5.3 ± 0.4	0.08
HDL cholesterol (mmol/L)	1.61 ± 0.36	1.29 ± 0.41	0.03
LDL cholesterol (mmol/L)	2.79 ± 0.69	2.82 ± 0.74	0.93
Triglyceride (mmol/L)	1.03 ± 0.42	1.30 ± 0.94	0.34
Free fatty acids (μmol/L)	430 ± 195	362 ± 169	0.34
Plasma BNP (pg/mL)	–	428 ± 365	n.a
**Echocardiography**			
LVEF (%)	–	30 ± 9	n.a
**Cardiopulmonary exercise testing**			
Peak VO_2_ (mL/kg/min)	33.5 ± 6.3	20.0 ± 4.6	< 0.01
AT VO_2_ (mL/kg/min)	18.1 ± 3.4	11.8 ± 2.1	< 0.01
**Medications**			
ACE inhibitors or ARBs	–	17 (100)	n.a
β blockers	–	16 (94)	n.a
Aldosterone antagonists	–	12 (71)	n.a
Statins	1 (8)	3 (18)	0.43

Values are mean ± SD or n (%). ACE: angiotensin-converting enzyme, ARB: angiotensin II receptor blocker, AT: anaerobic threshold, BNP: B-type natriuretic peptide, HbA1c: glycohemoglobin A1c, HDL: high-density lipoprotein, HOMA-IR: homeostasis assessment model of insulin resistance, LDL: low-density lipoprotein, LVEF: left ventricular ejection fraction, n.a.: not applicable, VO_2_: oxygen uptake.

### Systemic oxidative stress

**Figure 1:**
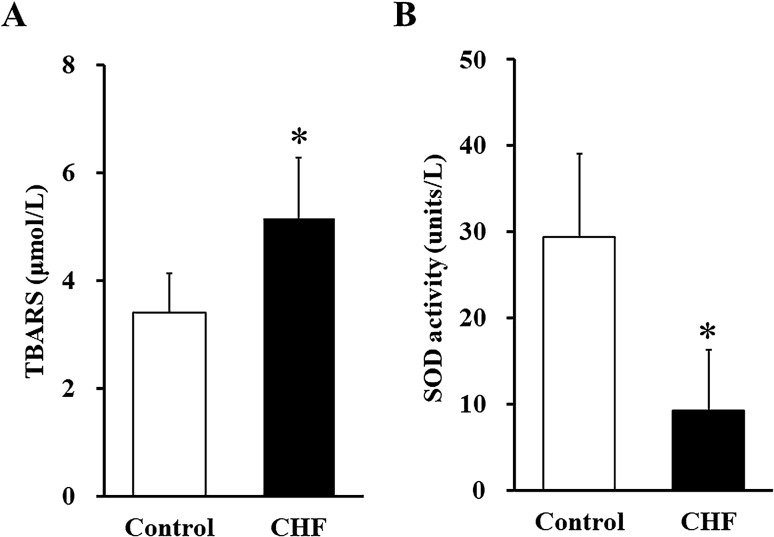
Systemic oxidative stress. (**A**) Serum TBARS (lipid peroxides) and (**B**) serum SOD activity (an antioxidant defense capacity). *White bars:* Control subjects (n = 13). *Black bars:* CHF patients (n = 17). Data are mean ± SD. *p < 0.01 vs. Control. SOD: superoxide dismutase, TBARS: thiobarbituric reactive substances.

The CHF patients' serum levels of thiobarbituric acid reactive substances (TBARS) were significantly higher (5.1 ± 1.1 vs. 3.4 ± 0.7 μmol/L, *p* < 0.01) (Fig. 1A) and their serum superoxide dismutase (SOD) activity was significantly lower compared to the control group (9.2 ± 7.1 vs. 29.4 ± 9.7 units/L, *p* < 0.01) (Fig. 1B), indicating both increased lipid peroxidation products and reduced antioxidant capacity in the CHF patients.

### Skeletal muscle energy metabolism

The magnetic resonance spectroscopy (MRS) data are summarized in Table 2. The one repetition maximum (1-RM) of plantar-flexion exercise was significantly lower in the CHF group than the control group (40 ± 6 vs. 35 ± 6 kg, *p* = 0.02), and the workload (20% 1RM) of plantar-flexion exercise was set in each participant. In the ^31^phosphorus (^31^P)-MRS study, the standardized phosphocreatine at rest (PCr_rest_) in the calf muscle was comparable between the CHF and control groups. However, the standardized PCr level at the lowest during the plantar-flexion exercise (PCr_lowest_) was significantly reduced in the CHF group, and as a result, the muscle PCr loss during the exercise was significantly greater in the CHF cohort, indicating impaired intramuscular high-energy phosphate energy metabolism in the CHF patients. In the ^1^proton (^1^H)-MRS study, the intramyocellular lipid (IMCL) content in the resting leg muscle was significantly increased in the CHF cohort, indicating impaired fatty acid metabolism in the skeletal muscle of the CHF patients.

**Table 2:** Skeletal muscle energy metabolism.

	Control (n = 13)	CHF (n = 17)	*p* value
^**31**^**P-MRS**			
Standardized PCr_rest_	0.88 ± 0.03	0.87 ± 0.02	0.39
Standardized PCr_lowest_	0.68 ± 0.09	0.48 ± 0.17	< 0.01
Muscle PCr loss	0.20 ± 0.08	0.39 ± 0.17	< 0.01
^**1**^**H-MRS**			
IMCL content, mmol/kg/wet weight	1.6 ± 0.9	4.0 ± 2.1	< 0.01

Values are mean ± SD. IMCL: intramyocellular lipid, PCr: phosphocreatine, PCr_rest_: PCr level at rest, PCr_lowest_: the lowest PCr level during plantar-flexion exercise with constant load of 20% one repetition maximum.

### The association between systemic oxidative stress and whole-body aerobic capacity

**Figure 2:**
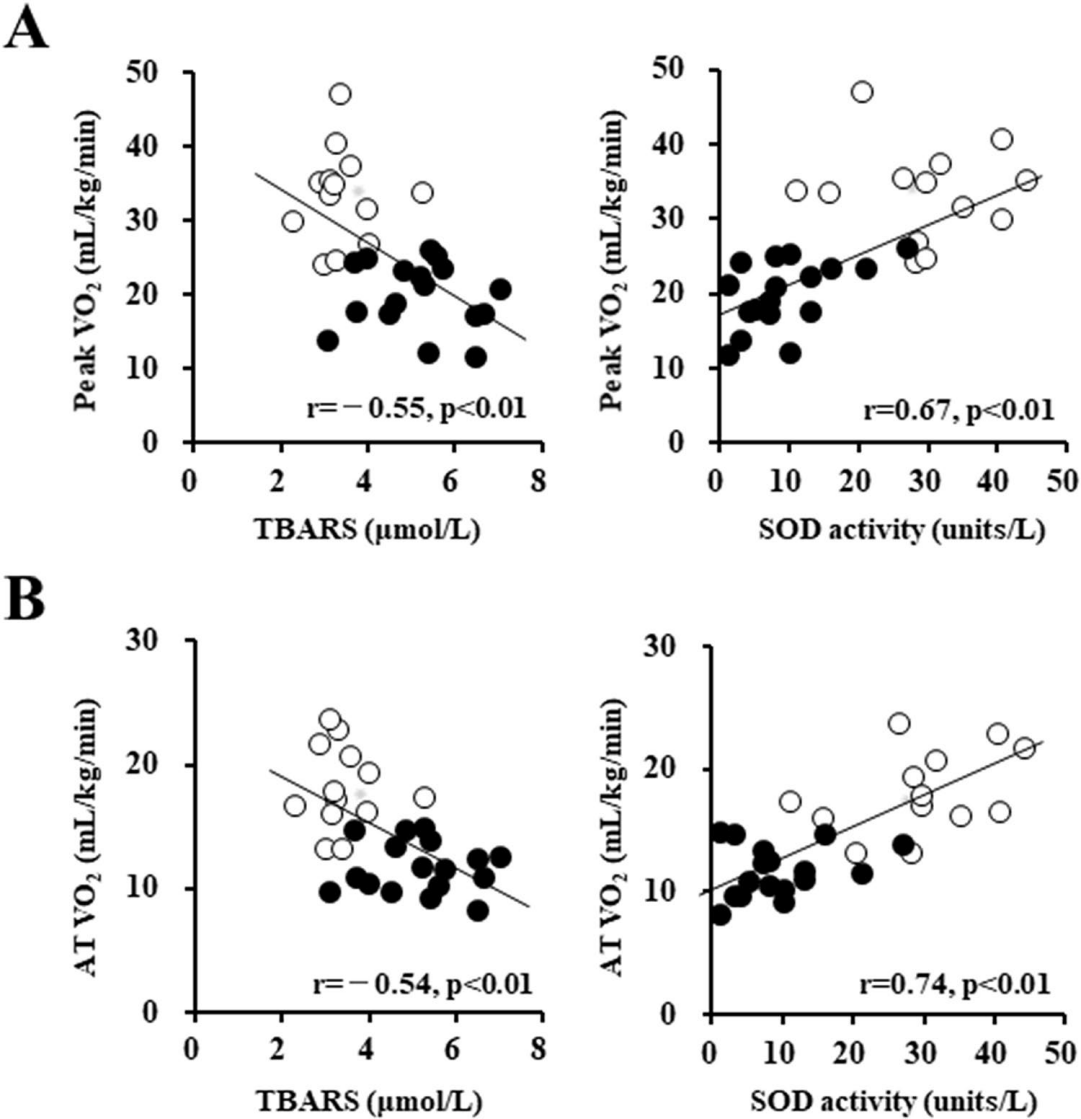
Linear relationship between systemic oxidative stress and whole-body aerobic capacity. (**A**) Systemic oxidative stress and peak VO_2_ and (**B**) systemic oxidative stress and AT VO_2_. *White circles:* Control subjects (n = 13). *Black circles:* CHF patients (n = 17). AT: anaerobic threshold, VO_2_: oxygen uptake.

The peak VO_2_ and the AT VO_2_ were inversely correlated with the serum TBARS level, whereas these two parameters were positively correlated with the serum SOD activity in the series of all participants (Fig. 2A,B), indicating that systemic oxidative stress is linked to reduced whole-body aerobic capacity. In addition, the peak VO_2_ was positively correlated with the serum SOD activity (r = 0.50, *p* = 0.04) in the CHF patients, but not with the patients' serum TBARS levels.

### The association between systemic oxidative stress and skeletal muscle energy metabolism

**Figure 3:**
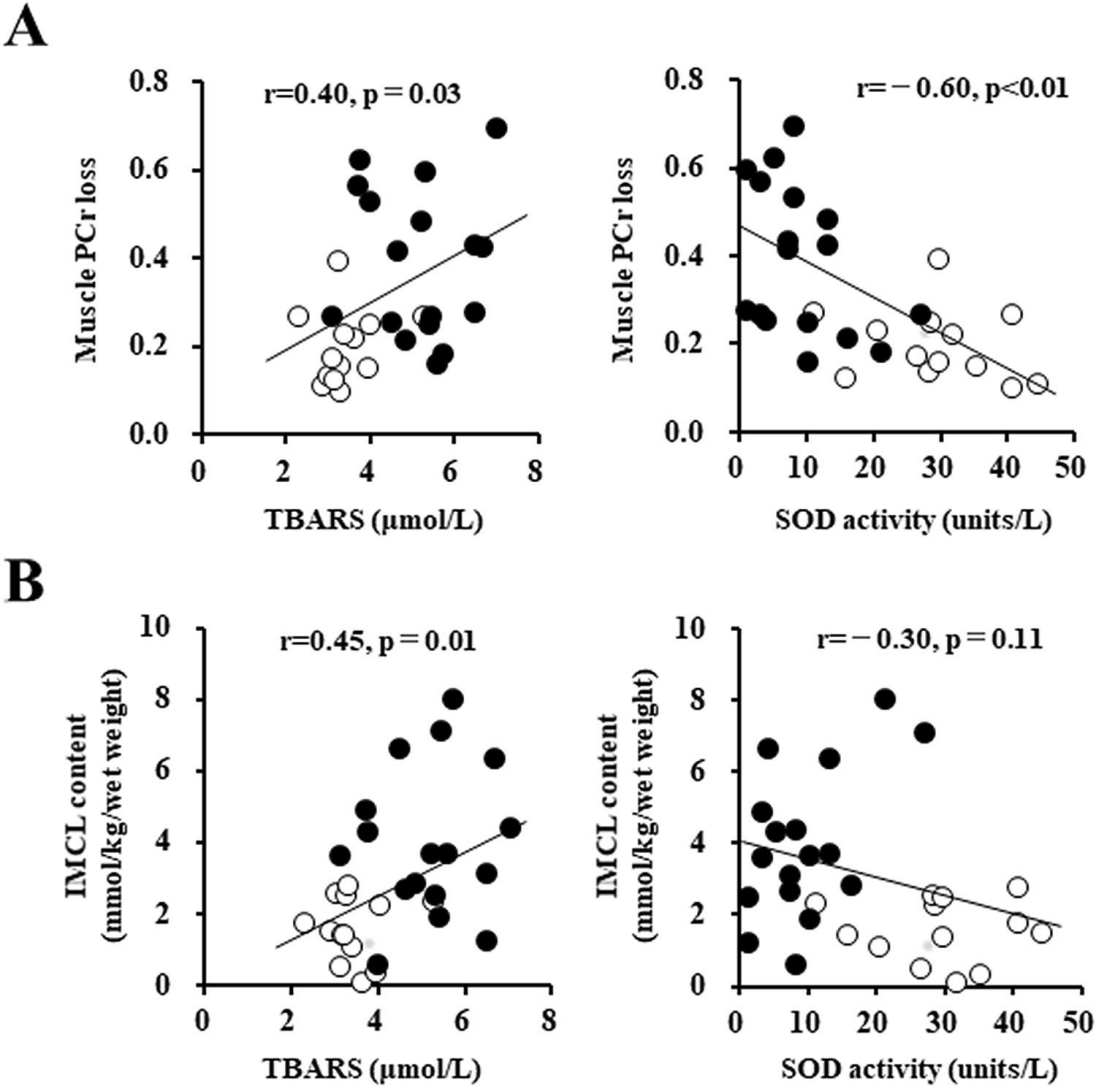
Linear relationship between systemic oxidative stress and skeletal muscle energy metabolism. (**A**) Systemic oxidative stress and muscle PCr loss during the plantar-flexion exercise and (**B**) systemic oxidative stress and IMCL content. *White circles:* Control subjects (n = 13). *Black circles:* CHF patients (n = 17). IMCL: intramyocellular lipid, PCr: phosphocreatine.

In the series of all participants, the muscle PCr loss during the plantar-flexion exercise had a positive correlation with the serum TBARS level and an inverse correlation with the serum SOD activity (Fig. 3A). The IMCL content also had a positive correlation with the serum TBARS level, although there was no significant correlation between the IMCL content and the serum SOD activity in the series of all participants (Fig. 3B).

## Discussion

The results of our analyses demonstrated that CHF patients had higher serum TBARS and lower serum activities of SOD, indicating increased systemic oxidative stress in CHF. The whole-body aerobic capacity characterized by the peak VO_2_ and the AT VO_2_ was decreased, and the skeletal muscle energy metabolism evaluated by the muscle PCr loss during exercise and the IMCL content was impaired in the CHF patients. Notably, both the higher serum TBARS and the lower serum SOD activity were linked to either impaired maximal and submaximal aerobic capacity (i.e., lower peak VO_2_ and AT VO_2_) or impaired intramuscular high-energy phosphate metabolism (i.e., increased muscle PCr loss during exercise). In addition, the higher serum TBARS levels were linked to accumulated IMCL content in the leg muscle. This study is the first to reveal associations between systemic oxidative stress and both exercise intolerance and skeletal muscle abnormalities in patients with CHF.

Systemic oxidative stress is reported to play a crucial role in disease progression in patients with CHF^8,10^. Previous studies have shown that several circulating oxidative stress markers may predict adverse clinical events including all-cause death and hospitalization in CHF patients^9,11^. Because exercise intolerance is related to the morbidity and mortality of CHF patients, our present findings of associations of systemic oxidative stress with lowered whole-body aerobic capacity and impaired skeletal muscle energy metabolism may contribute to the understanding of the potential roles and effects of enhanced systemic oxidative stress in the progression of heart failure.

There are some possible mechanisms by which systemic oxidative stress is enhanced in CHF patients. First, excessive ROS production in a failing heart may increase the level of circulating free radicals. Clinical and experimental studies have demonstrated enhanced ROS production and oxidative stress in the failing heart^12–15^. The evidence of an increased number of ROS-loaded blood cells in the coronary sinus compared to that in the peripheral veins in patients with CHF suggests an increased rate of circulating blood cells exposed by myocardial ROS when they pass through the heart^16^. Indeed, we have observed that mitochondrial ROS in circulating blood cells were elevated in symptomatic CHF patients in relation to systemic oxidative stress^10^.

Second, skeletal muscles can be a source of ROS in CHF, which supports the hypothesis that increased myocellular ROS directly impair skeletal muscle energy metabolism. We and others have shown that HF-model rodents had enhanced mitochondrial oxidative stress in their skeletal muscle^17,18^. We also demonstrated that one-leg immobilization for 2 weeks induced excessive ROS production accompanied by impaired mitochondrial respiratory capacity in the immobilized leg muscles of humans^19^. Accordingly, physical inactivity as well as the intrinsic impairment of skeletal muscle function under the condition of CHF seems to contribute to greater ROS production in the skeletal muscles, leading to the enhanced systemic oxidative stress in CHF patients.

Finally, physical inactivity itself may reduce the body's systemic antioxidant defense capacity^20^. Although we did not evaluate the participants' daily physical activity in the present study, exercise intolerance can induce a poor quality of life with a reduction of physical activity, which may result in lowered serum SOD activity in CHF patients. Further studies are needed to clarify the mechanisms underlying the enhanced systemic oxidative stress in CHF patients.

We investigated skeletal muscle energy metabolism by using MRS, which is a well-validated and noninvasive method. We measured the PCr loss in the calf muscle during the plantar-flexion exercise with the constant load of 20% 1-RM to assess the intramuscular high-energy phosphate metabolism using ^31^P-MRS. The PCr is an important energy source and works as an energy buffer, because the PCr can be promptly converted into ATP to maintain the ATP at a constant level during exercise. Since exercise with the load of 20% 1-RM is low-intensity exercise without a decrease in muscle pH (i.e., aerobic exercise), our finding that the muscle PCr loss during the plantar-flexion exercise was increased in the CHF patients might indicate impaired muscle oxidative metabolism.

We also measured the IMCL content in the resting leg muscle by ^1^H-MRS. The IMCL content is commonly defined by an imbalance between the uptake of fatty acids into the muscle and the intramuscular fatty acid oxidation. In this study, the free fatty acid levels were comparable between the CHF patients and control subjects, and thus the accumulated IMCL in the CHF patients might be attributable mainly to the impaired fatty acid oxidation in the skeletal muscle.

Heart failure-related myopathy characterized by impaired skeletal muscle energy metabolism and altered muscle structure (e.g., muscle atrophy and muscle fiber type switch) is a major contributor to the exercise intolerance of CHF patients^5,6,21^. It has been shown that exercise training improves skeletal muscle function and augments systemic and intramuscular antioxidant capacity in patients with CHF^22^. Accordingly, exercise training may be beneficial for the restoration of the redox balance as well as the improvement of cardiorespiratory fitness in these patients.

There are some study limitations to consider. First, the number of participants was small (n = 30). Second, the correlations between systemic oxidative stress and exercise intolerance or impaired skeletal muscle energy metabolism were not significant except for the correlation between the serum SOD activity and peak VO_2_ when we analyzed only the data of the CHF patients. Because the range of parameters of exercise capacity and skeletal muscle energy metabolism within the CHF group was small, we could not detect a significant correlation. Third, we could not identify the causality of the relationships between systemic oxidative stress and exercise intolerance or skeletal muscle dysfunction. Further studies with larger numbers of participants are necessary to clarify the causal relationships among these parameters.

In summary, this study demonstrated for the first time that systemic oxidative stress was related to exercise intolerance and skeletal muscle abnormalities in patients with CHF. Our findings suggest that restoration of the redox balance is beneficial in the treatment of CHF.

## Methods

### Participants

Thirty male participants were recruited at Hokkaido University Hospital: 17 stable CHF patients who had a history of hospitalization due to worsening HF defined by the Framingham Criteria^23^ and 13 age-matched healthy subjects as a control group. We excluded patients who had difficulty performing maximal exercise due to a physical problem including orthopedic disease, stroke, severe pulmonary disease, and peripheral artery disease, and patients with diabetes or severe chronic kidney disease. Patients who had been assessed as being NYHA functional class IV were also excluded. The present report is part of an MRS study targeting lifestyle disorders and cardiovascular diseases, and thus some of the data used herein were obtained from the same patients whose data were published previously but in a different context^4^. The protocol was approved by the Medical Ethics Committee of Hokkaido University Hospital, and written informed consent was obtained from each participant before the study. All investigations conformed to the principles outlined in the Declaration of Helsinki.

### Study protocol

The participants underwent blood tests for the analyses of blood biochemistry and systemic oxidative stress after a 10-h overnight fast, followed by clinical and anthropometric measurements, echocardiography, and a ^1^H-MRS study to measure the IMCL content in the resting leg muscle. The participants also underwent cardiopulmonary exercise testing (CPET) to assess their exercise capacity. On another day, a ^31^P-MRS study was conducted to assess the high-energy phosphate metabolism in the calf muscle during plantar-flexion exercise.

### Blood testing

Fasting blood glucose, plasma insulin, HbA1c, HDL-cholesterol, LDL-cholesterol, triglyceride, and free fatty acids were measured in all participants, and the plasma levels of brain natriuretic peptide (BNP) were measured in only the CHF patients by routine in-house analyses. The HOMA-IR was calculated as described^24^. To assess systemic oxidative stress, we measured the serum TBARS and the enzymatic activity of SOD by conducting fluorometric analyses in all participants as described^25^.

### Echocardiography

Cardiac function was evaluated by echocardiography in the CHF patients as described^26^. The left ventricular ejection fraction (LVEF) was measured from apical four- and two-chamber images using the biplane method of disks.

### CPET

The participants performed cardiopulmonary exercise testing (CPET) using an upright bicycle ergometer (Aerobike 75XLII; Combi Wellness, Tokyo) with a ramp protocol (10–25 watts/min) as described^27,28^. A respiratory gas analysis was simultaneously performed with a breath-by-breath apparatus (Aeromonitor AE-300S; Minato Medical Science, Osaka, Japan). The peak VO_2_ was defined as the VO_2_ attained at the maximal point during symptom-limited incremental exercise (i.e., at the point of leveling-off despite the increased workload). The AT VO_2_ was determined by the V-slope method by at least two CPET experts.

### The ^31^P-MRS study

Before the study, the 1-RM of plantar-flexion exercise, i.e., the maximal weight of exercise that can be done only once, was determined with the participant in the supine position as described^4,28^. After a ≥ 30-min rest, the high-energy phosphate metabolism in the calf muscle was measured during plantar flexion exercise in the supine position on the original apparatus equipped with a 1.5-T whole-body scanner system (Magnetom Vision VB33G, Siemens, Erlangen, Germany) using ^31^P-MRS as described^4,28^. The exercise protocol was a constant load of 20% 1-RM at the pace of 40 times/min for 4 min. Usually, immediately after the initiation of the plantar-flexion exercise, the PCr level in the calf muscle started to decrease and was finally stabilized within a few minutes. The PCr was standardized as [PCr] / ([PCr] + [Pi]) on the basis of the notion that [PCr] + [Pi] is constantly equal both at rest and during exercise, where [PCr] indicates the concentration of PCr and [Pi] indicates the concentration of inorganic phosphate (Pi). The degree of PCr change (i.e., the PCr loss) during exercise was calculated as PCr loss = standardized PCr_rest_ − standardized PCr_lowest_.

### The ^1^H-MRS study

We measured the IMCL content in the resting tibialis anterior muscle at the level of the muscle belly of the calf using ^1^H-MRS on a 1.5-T whole-body scanner system (Signa Horizon LX, GE Medical Systems, Milwaukee, WI) as described^4,28^.

### Statistical analyses

Data are expressed as the mean ± standard deviation (SD) or n (%). We used Student's *t-*test for continuous variables and the χ^2^ test for categorical variables to compare the data between the CHF and control groups. We examined correlations by performing a linear regression analysis using Pearson's correlation coefficient. Statistical analyses were performed using GraphPad Prism ver. 8 (GraphPad Software, San Diego, CA), and significance was defined as *p* < 0.05.
